# p53 status as effect modifier of the association between pre-treatment fasting glucose and breast cancer outcomes in non diabetic, HER2 positive patients treated with trastuzumab

**DOI:** 10.18632/oncotarget.2060

**Published:** 2014-06-05

**Authors:** Patrizia Vici, Francesca Sperati, Marcello Maugeri-Saccà, Elisa Melucci, Anna Di Benedetto, Luigi Di Lauro, Laura Pizzuti, Domenico Sergi, Irene Terrenato, Luca Esposito, Carmelina Antonella Iannuzzi, Raffaella Pasquale, Claudio Botti, Barbara Fuhrman, Antonio Giordano, Marcella Mottolese, Maddalena Barba

**Affiliations:** ^1^ Division of Medical Oncology B, Regina Elena National Cancer Institute, Rome, Italy; ^2^ Biostatistics-Scientific Direction, Regina Elena National Cancer Institute, Rome, Italy; ^3^ Division of Medical Oncology B-Scientific Direction, Regina Elena National Cancer Institute, Rome, Italy; ^4^ Department of Pathology, Regina Elena National Cancer Institute, Rome, Italy; ^5^ Center for Oncologic Research of Mercogliano (CROM), Avellino, Italy; ^6^ Oncology Research Centre of Mercogliano (CROM), G. Pascale Foundation National Cancer Institute, Naples, Italy; ^7^ Department of Surgery, Regina Elena National Cancer Institute, Rome, Italy; ^8^ Department of Epidemiology, University of Arkansas for Medical Sciences, Arkansas, USA; ^9^ Sbarro Institute for Cancer Research and Molecular Medicine and Center of Biotechnology, College of Science and Technology Temple University, Philadelphia, USA

**Keywords:** p53 status, fasting glucose, HER2 positive breast cancer, trastuzumab.

## Abstract

Mounting evidence supports the role of p53 in metabolic processes involved in breast carcinogenesis. We investigated whether p53 status affects the association of pre-treatment fasting glucose with treatment outcomes in 106 non diabetic, HER2 positive breast cancer patients treated with trastuzumab. p53 status was validated against gene sequencing of selected codons in 49 patients. The Kaplan–Meier method and log rank test were used to compare survival by categories of fasting glucose in the overall population and separate settings. Cox models included age and body mass index. Direct sequencing confirmed the lack of mutations in 73.7% of p53 negative patients and their presence in 53.3% of p53 positive cases. At 66 months, 88.3% of patients with glucose ≤ 89.0 mg/dl (median value) did not experiment disease progression compared with 70.0% in the highest category (*p*=*0.034*), with glucose being an independent predictor (*p*=*0.046*). Stratified analysis confirmed this association in p53 negative patients only (p=0.01). In the early setting, data suggested longer disease free survival in p53 negative patients in the lowest glucose category (p=0.053). In our study, p53 status acted as effect modifier of the investigated association. This may help differentiate target sub-groups and affect outcomes interpretation in similarly characterized patients.

## INTRODUCTION

Over the past three decades, considerable and consistent evidence has firmly established the role of p53 in protecting genetic stability by constraining proliferation of malignant cells, motility and viability of abnormal or stress-exposed cells [1]. In recent years, accumulating evidence has provided support to the key role of p53 in homeostatic regulation of metabolic processes involved in carcinogenesis, including pathways related to glucose metabolism. In the context of cell adaptation favoring carcinogenesis, increasing attention has been paid to the prevalence of aerobic glycolytic “Warburg” metabolism, i.e., metabolic cell reprogramming for producing of energy throughout glycolysis even under normal oxygen concentrations. The loss of p53 generally favors the shift from aerobic respiration to (aerobic) glycolysis. However, p53 specific action on cancer metabolism might be driven and ultimately established by a plethora of still poorly characterized factors and inadequately explored pathways [2-4].

Research in breast cancer has lately allowed to characterize molecular features that have increasingly become the targets for novel therapeutic interventions. Trastuzumab has revolutionized the treatment of patients with HER2 positive breast cancer. However, approximately 10% of these patients develop a distant recurrence following adjuvant trastuzumab-based chemotherapy, and all patients with metastatic disease eventually develop disease progression. The identification of biomarkers that are likely to predict which patients will achieve the best response to this agent represents a major challenge for oncologists [5].

Extensive pre-clinical and epidemiologic studies substantiate the link between factors related to glucose metabolism and breast carcinogenesis. Redundancy and crosstalks among the numerous signaling networks that regulate growth and survival in epithelial tumours are present. Laboratory evidence supports the interaction between factors related to glucose metabolism [e.g. fasting glucose, insulin-like growth factors (IGFs)] and members of the erbB tyrosine kinase family, such as HER2 [6, 7]. Moreover, in prospective cohort studies, diabetes and increased glucose levels have been consistently associated with an increased risk of breast cancer development [8-10].

We have recently focused on the predictive role of pre-treatment fasting glucose in the development of resistance to trastuzumab in non diabetic, HER2 positive breast cancer patients. Results from our study showed significantly longer time to disease progression in patients with lower levels of pre-treatment fasting glucose compared to the highest tertiles [11]. In the present study, we sought to investigate whether p53 immunohistochemical staining patterns might affect the association between the biomarker of interest and treatment outcomes in a well characterized subset of the original study population.

## RESULTS

The characteristics of the 106 study participants included in our analysis are shown in Table 1. The median follow up was 28.8 months (1-128.3). Thirteen patients (12.2%) were lost to follow up. Included variables are related to the patients' demographics, menopausal status and metabolic profile (i.e., BMI, fasting glucose at baseline and follow up) as well as to breast cancer features at diagnosis (i.e., stage, molecular characteristics).

**Table 1:** Characteristics of the study participants (N=106)

Age at Cancer Diagnosis* (mean, ±SD)		48,1	±10,9
Menopausal Status (n, %)	Premenopausal	55	51,9
	Postmenopausal	51	48,1
BMI at Baseline^§^ (mean, ±SD)		25,0	±4,1
Stage at Cancer Diagnosis (n, %)	I	62	58,5
(TNM)	II	11	10,4
	III	8	7,5
	IV	25	23,6
BMI at Baseline^§^ (mean, ±SD)		25,0	±4,1
Fasting Glucose at Baseline ^#^ (mean, ±SD)		91,3	±11,2
Fasting Glucose at Follow-up^#^ (mean, ±SD)		92,8	±16,5
p53 (n, %)	Negative	53	50,0
	Positive	53	50,0
Estrogen Receptor (ER) (n, %)	<10	51	48,1
	≥10	55	51,9
Progesteron Receptor (PgR) (n, %)	<10	56	52,8
	≥10	50	47,2
Ki67 (n, %)	≤15	35	33,0
	>15	71	67,0
Bcl-2 (n, %)	≤30	72	67,9
	>30	34	32,1

* in years,

** in centimeters,

*** in kilograms (Kg),

§ Body Mass Index in m^2^ / Kg,

# milligrams/dl

In Table 2, our study participants were compared by categories of pre-treatment fasting glucose defined upon the median value (i.e. 89.0 mg/dl). No significant differences were observed across the categories examined, neither by general characteristics nor by molecular features.

**Table 2:** Characteristics of the study participants by categories of pre-treatment fasting glucose defined upon the median value (89,0 mg/dl) (N=106)

		Fasting Glucose at Baseline#		
		≤89	>89	p-value°
Age at Cancer Diagnosis* (mean±sd)		46,1±11,5	50,1±9,9	0,064
Menopausal Status n (%)	Premenopausal	30 (54,5)	25 (45,5)	0,331
	Postmenopausal	23 (45,1)	28 (54,9)	
BMI at Baseline^§^ (mean±sd)		24,4±3,7	25,6±4,4	0,129
Stage at Cancer Diagnosis n (%)	I	30 (48,4)	32 (51,6)	0,874
(TNM)	II	5 (45,5)	6 (54,5)	
	III	5 (62,5)	3 (37,5)	
	IV	13 (52,0)	12 (48,0)	
p53 n (%)	Negative	27 (50,9)	26 (49,1)	0,846
	Positive	26 (49,1)	27 (50,9)	
Estrogen Receptor (ER) n (%)	<10	26 (51,0)	25 (49,0)	0,846
	≥10	27 (49,1)	28 (50,9)	
Progesterone Receptor (PgR) n (%)	<10	25 (44,6)	31 (55,4)	0,243
	≥10	28 (56,0)	22 (44,0)	
Ki67 n (%)	≤15	14 (40,0)	21 (60,0)	0,148
	>15	39 (54,9)	32 (45,1)	
Bcl-2 n (%)	≤30	40 (55,6)	32 (44,4)	0,096
	>30	13 (38,2)	21 (61,8)	

* in years,

** in centimeters,

*** in kilograms (Kg),

§ Body Mass Index in m2 / Kg,

# Mg/dl,

° Comparisons were performed with the Pearson's Chi-Square Test for the categorical variables and with T-Student test for continuous variables.

Cancer patient characteristics by p53 status appear in Table 3. Women testing negative at immunostaining showed a significantly lower percentage of ki67 expression compared with positive cases (42.3% vs. 57.7%, p= 0.023). No further relevant differences emerged.

**Table 3:** Study participants' characteristics by p53 status immunohistochemical staining pattern. (N=106)

		p53 status		
		p53-negative	p53-positive	p-value°
Age at Cancer Diagnosis* (mean±sd)		48,2±9,8	48,0±12,0	0,954
Menopausal Status n (%)	Premenopausal	27 (49,1)	28 (50,9)	0,846
	Postmenopausal	26 (51,0)	25 (49,0)	
Marital Status n (%)	Married	37 (52,1)	34 (47,9)	0,986
	Separated	3 (50,0)	3 (50,0)	
	Single	8 (47,1)	9 (52,9)	
	Widowed	2 (50,0)	2 (50,0)	
Smoking Status n (%)	Yes	9 (45,0)	11 (55,0)	0,611
	No	35 (51,5)	33 (48,5)	
BMI at Baseline^§^ (mean±sd)		25,0±3,3	25,0±4,8	0,986
Stage at Cancer Diagnosis n (%)	I	31 (50,0)	31 (50,0)	0,378
(TNM)	II	6 (54,5)	5 (45,5)	
	III	6 (75,0)	2 (25,0)	
	IV	10 (40,0)	15 (60,0)	
Fasting Glucose at Baseline (mean±sd)		91,8±11,4	90,7±11,1	0,625
Fasting Glucose at Follow-up (mean±sd)		94,4±11,4	91,2±20,5	0,329
Estrogen Receptor (ER) n (%)	<10	22 (43,1)	29 (56,9)	0,174
	≥10	31 (56,4)	24 (43,6)	
Progesterone Receptor (PgR) n (%)	<10	27 (48,2)	29 (51,8)	0,697
	≥10	26 (52,0)	24 (48,0)	
Ki67 n (%)	≤15	23 (65,7)	12 (34,3)	0,023
	>15	30 (42,3)	41 (57,7)	
Bcl-2 n (%)	≤30	34 (47,2)	38 (52,8)	0,405
	>30	19 (55,9)	15 (44,1)	

* in years,

** in centimeters,

*** in kilograms (Kg),

§ Body Mass Index in m2 / Kg,

° Comparisons were performed with the Pearson's Chi-Square Test for the categorical variables and with T-Student test for continuous variables.

**Figure 1:**
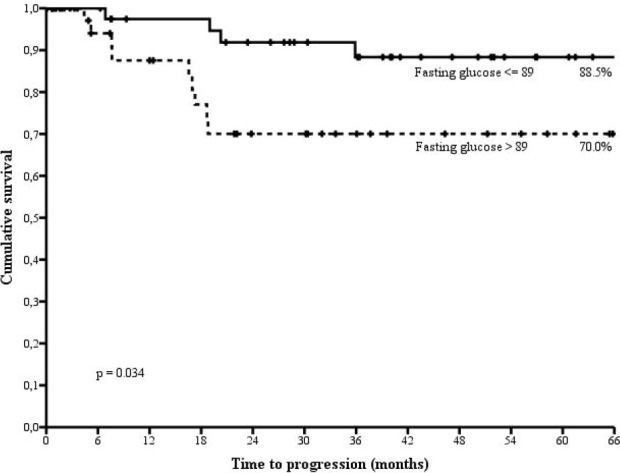
Time to disease progression by categories of fasting glucose defined upon the median value (89.0 mg/dl) in non diabetic, HER2 positive breast cancer patients treated with trastuzumab

**Figure 2:**
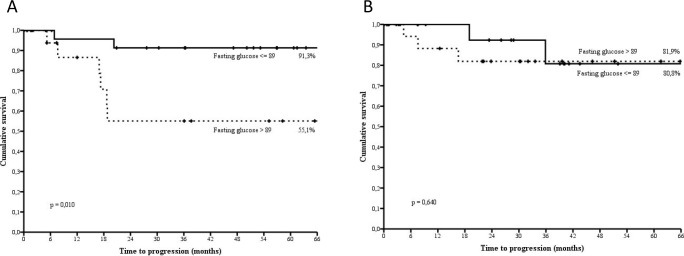
Time to disease progression by categories of fasting glucose defined upon the median value (89.0 mg/dl) in p53 negative (2a) and p53 positive (2b) non diabetic, HER2 positive breast cancer patientstreated with trastuzumab

Figure 1 shows time to disease progression through categories defined upon the median value of pre-treatment fasting glucose. In the overall population, at 66 months, 88.3% of cancer patients in the lowest glucose category did not experiment disease progression compared with 70.0% in the highest category (p= 0.034). We then stratified survival data by p53 status (Figure 2a-b). In women testing negative at immunostaining, the association between circulating levels of pre-treatment fasting glucose was confirmed and statistically reinforced. At 66 months, 91.3% of breast cancer patients whose fasting glucose was below the median value showed no evidence of disease progression, compared with 55.1% in the highest glucose category (p= 0.010) (Figure 2a). Conversely, the assessment of the association between pre-treatment fasting glucose and treatment outcome in patients testing positive at immunostaining revealed no significant differences (Figure 2b).

In multivariate analysis including age at cancer diagnosis and BMI as covariates, the predictive role of pre-treatment fasting glucose on time to disease progression was confirmed, (HR 6.35, 95%CI 1.04-38.90, *p= 0.046*). The Cox model testing the interaction between p53 status and BMI produced no significant results (p= 0.466) (Table 4).

**Table 4:** Cox proportional hazard models of factors associated with time to disease progression in non diabetic, HER2 positive breast cancer patients (N=106)

		Univariate			Multivariate		
		HR	95CI%	p-value	HR	95CI%	p-value
Age at diagnosis°		1,00	0,95-1,05	0,924	0,96	0,87-1,06	0,408
Fasting glucose at baseline#	≤89	1			1		
	>89	3,33	1,02-10,84	0,046	6,85	1,02-45,90	0,047
BMI§		1,01	0,89-1,13	0,934	1,02	0,89-1,18	0,743
P53	Negative	1					
	Positive	0,77	0,25-2,35	0,644			
P53*BMI§		0,98	0,93-1,03	0,466	0,98	0,93-1,03	0,419
P53*subtype		1,66	0,51-5,41	0,397			

° in years,

# Mg/dl,

§ m^2^/Kg.

**Figure 3:**
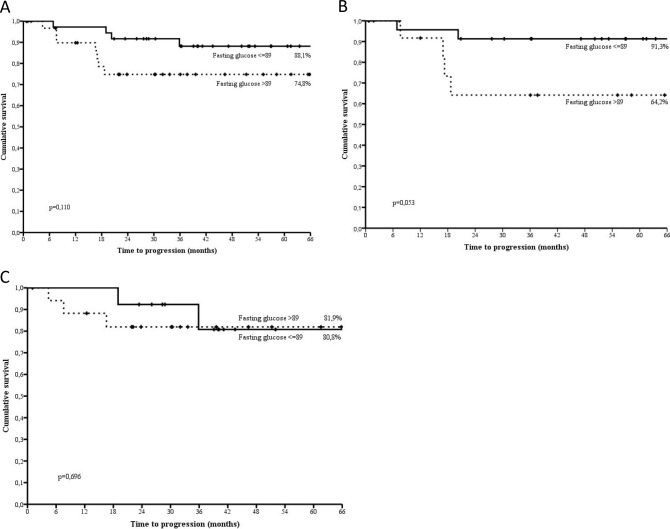
Disease free survival by categories of fasting glucose defined upon the median value (89.0 mg/dl) in the overall subset (3a), p53 negative (3b) and p53 positive (3c) non diabetic, HER2 positive breast cancer patients treated with trastuzumab

Within the early setting, which included 81 women who had received trastuzumab as neoadjuvant or adjuvant treatment, survival estimates did not significantly differed by glucose category (p=0.110). However, when stratifying by p53 status, data were suggestive of longer disease free survival for women with glucose below the median exclusively in the p53 negative subgroup (p=0.053) (Figure 3a-b-c). Of the 25 patients with stage IV breast cancer at diagnosis, only two contributed events to our analysis and were both located in the highest category of fasting glucose. This made further analysis in the advanced setting unfeasible.

Among the 49 breast cancer patients for whom p53 immunohistochemical status was validated against gene sequencing, 18 exhibited at least one missense mutations (about 37%). More specifically, we observed 17 Arginina-Serine and 1 Arginine-Cysteine missense mutations in codon 249 and 273, respectively. Overall, results from gene sequencing confirmed p53 status as immunohistochemically assessed in 30 patients out of 49 (30/49), with the raw agreement being 61.2%. More specifically, we did not observe somatic mutations in 14/19 cases with negative immunostaining for p53 (raw agreement: 73.7%), while mutations were identified in 16/30 p53 positive cases (raw agreement: 53.3%).

## DISCUSSION

In the present study, a set of analyses on a relatively small, though very well characterized, group of 106 non diabetic, HER2 positive breast cancer patients treated with trastuzumab confirmed our previous findings concerning the predictive role of pre-treatment fasting glucose on treatment outcomes. Stratification of survival analyses by p53 status allowed the distinction between two different subpopulations. In p53 negative patients, the association between fasting glucose and time to disease progression was confirmed and the level of statistical significance increased. This same association was not verified in women testing positive at p53 immunostaining. Survival data stratified by setting provided evidence of borderline significant effect modification associated with p53 status in the early setting.

Findings linking a systemic biomarker related to glucose metabolism, i.e., pre-treatment fasting glucose, to breast cancer outcomes are not novel. Indeed, large and consistent evidence of better outcomes in breast cancer patients with lower levels of fasting glucose compared to the highest categories have come from a number of observational studies [11,12-14]. Similarly, previous studies have largely, though not always consistently, documented the prognostic role of p53 in breast cancer [15-18]. Conversely, the predictive role of immunohistochemically-assessed p53 status on treatment outcomes in patients receiving trastuzumab has been poorly documented and results do not seem to be supportive [19]. However, to the best of our knowledge, the hypothesis of a role of p53 as effect modifier of the association between pre-treatment fasting glucose and breast cancer outcomes has neither been formulated nor (been) tested before in the clinical setting. Though still highly speculative, characterization of p53 status might contribute to better characterize the target population and interpret treatment outcomes for interventions based on trastuzumab administration in non diabetic patients and inform decisions on co-interventions targeting glucose metabolism. In case of confirmatory findings from future studies in similarly characterized patients, it is conceivable that lifestyle-related and pharmacological co-interventions acting on glucose metabolism in women receiving trastuzumab might possibly increase treatment efficacy and translate into improved survival outcomes.

Immunohistochemical analysis of p53 expression is widely accepted as a surrogate for mutational analysis [20-23]. Yet, genomic DNA sequencing is the most reliable technique in detecting gene mutations. However, within the subset examined (i.e., 49 randomly extracted breast cancer patients), raw agreement between results from immunohistochemestry and sequencing was quite satisfactory in the p53 positive group (53.3%) and high in the p53 negative group (73.7%). Several possible explanations might have contributed to having not observed a perfect correlation between immunohistochemical expression and *TP53* mutations. First, we examined a limited number of codons, namely, 175, 245, 248, 249 and 273, that are likely to identify most, though not all, the mutated loci. Second, genetic assessment of *TP53* was performed on PETT at the time of our study, while immunostaining was defined on distinct tissues sampled at cancer diagnosis. Tumour heterogeneity might have at least partly contributed to discrepancies in our results [24]. Differences in raw agreement between the two subgroups (i.e., p53 positive and negative) might also be interpreted in light of the relatively low percentage of HER2 Enriched (HER2E) subtype in p53 positive cases (43.3%, i.e., 13/30) and (relatively) higher frequency of Luminal B subtype in the p53 negative subgroup (63.2%, i.e., 12/19). Indeed, *TP53* mutations are quite often represented in the HER2E subtype (72%), while are less often encountered in Luminal B cancers (29%) [25].

Evidence supporting the role of p53 on patient energy balance has recently emerged from an observational study of 1060 colorectal cancers. In nonobese patients (BMI<30 kg/m^2^) with p53 negative tumours, specific mortality increased significantly. On the contrary, p53 positivity was not associated with patient survival outcomes in obese women (BMI≥30 kg/m^2^) [26]. These data seem to indicate that metabolic abnormalities and excess energy balance underlying obesity might be specifically detrimental in patients with p53 negative tumors. In this and a previous study of ours, we included BMI at baseline (prior to any treatment administration) in Cox models testing the independent role of fasting glucose on treatment outcome. Based on the recent findings from Morikawa, we have now added an interaction term between BMI and p53 status. Both the models produced not significant results (p= 0.917 and 0.601 for BMI and p53*BMI, respectively).

The effects of the burden of disease, as expressed by cancer stage at diagnosis, on treatment outcomes is intuitive. In the early setting, we could not observe significantly different survival estimates until stratification by p53 status was introduced. This may somewhat more strongly support the role of p53 as effect modifier of the association of interest. Indeed, notwithstanding the reduced statistical power due to focusing on 81 patients only, stratification by p53 status highlighted a borderline significant advantage in terms of disease free survival for women in the lowest category of fasting glucose. Unfortunately, survival data related to the advanced setting were poorly informative. Indeed, due to the insufficient length of follow up at study closure, the number of patients contributing events to our study was extremely low (i.e., 2).

At the cellular level, exposure to high fasting glucose, particularly when occurring on a chronic basis, introduces a source of metabolic disturbance which is particularly well characterized in diabetic patients [27]. There is substantial evidence on the role of p53 as a key mediator in cell metabolic adaptation. Activation of p53 induced by metabolic stress is mainly, though not exclusively, driven by AMPK-dependent phosphorylation via LKB1 and mostly influenced by mTOR [28-33]. p53 and mTOR are linked by extensive cross talk. p53 governs vital cell functions including apoptosis, cell cycle arrest and senescence. Its anti-aging effects are mediated by growth-promoting pathways such as PI3K/mTOR, which converts cell cycle arrest into senescence. p53 can exert inhibitory effects on the mTOR pathway, as shown by decreased activity of mTOR in tissues of p53−/− mice [34-35]. At the metabolic level, p53 mediates several effects including inhibition of glycolysis and stimulation of fatty acids. mTOR is a nutrient-sensing pathway [35-37]. In mice fed ad libitum, mTOR is continuously activated, whereas calorie restriction decreases mTOR activation and partially relieves the loss of p53. Administration of the mTOR inhibitor Rapamycine delays tumour onset in cancer-prone p53^+/−^ and p53^−/−^mice [38]. In addition, there is large and consistent evidence supporting a multiple level interaction between p53- and the HER2-mediated signaling, also involving the p53 downstream CKI p21, p53 inhibitor Mdm2 and the tumour suppressor PTEN.

However, this study does have some limitations. We analyzed data from a relatively limited sample. Sample size limitations became even more evident when working on subgroups defined upon breast cancer setting. We are fully aware of the need of exploring our hypothesis in larger, ad hoc designed and possibly prospective studies which may adequately represent differences by setting. However, patients included in our analysis were very well characterized by molecular features. In addition, our laboratories' accreditation to ISOs 9001 together with the availability of control protocols for HER2 characterization, increase our confidence in data quality [34] [39].

Novelty is probably the main strength of our study. Our first time finding provides support to the role of p53 status as effect modifier of the association between pre-treatment fasting glucose and treatment outcomes in a well specified molecular subtype of breast cancer, i.e., HER2 positive, in non diabetic patients treated with trastuzumab. Given the current availability of low cost interventions related to glucose metabolism (e.g. life style, metformin), this pipeline might rapidly lead to future studies with innovative hints in terms of increasingly well defined target population and efficacy outcomes. The validation study performed on a subset of the 106 patients included along with the satisfactory degree of agreement between results from p53 immunostaining and TP53 sequencing, particularly in p53 negative patients, increases the reliability of our results.

In summary, we provide novel evidence on a widely used biomarker, namely, p53 immunostaining, as a bridging element between a systemic indicator of the glycemic body asset (fasting glucose) and treatment outcomes in a relatively small historic cohort of non diabetic, HER2 positive breast cancer patients treated with trastuzumab. The shift from investigating an association whose terms have long been somewhat generic to shaping study design and data analysis according to specific patient- and disease-related features might lead to increased specificity and, ultimately, efficacy of interventions based on trastuzumab administration and co-interventions targeting glucose metabolism in breast cancer.

## METHODS

### Study Participants and Settings

Included patients were 106 non diabetic, HER2 positive breast cancer patients treated with trastuzumab for whom data on p53 immunohistochemical staining patterns were available. These patients were a subset of a larger historic cohort of 202 breast cancers included in a previous analysis. Extensive details concerning study participants and setting for the main study were reported elsewhere [11]. In brief, breast cancer patients were considered suitable for inclusion if they had received trastuzumab according to the indications released by the US Food and Drug Administration (FDA) (http://www.fda.gov/Drugs/DevelopmentApprovalProcess/HowDrugsareDevelopedandApproved/ApprovalApplications/TherapeuticBiologicApplications/ucm080591.htm; http://www.cancer.gov/cancertopics/druginfo/fda-trastuzumab). We posed no restrictions by breast cancer setting, neither did we in reference to the administration schedule. We excluded patients diagnosed with either type I or type II diabetes based on laboratory data and those whose pre-treatment fasting glucose was equal to or higher than 126 mg/dl [35] [40].

### Data Retrieving

For each patient a specifically trained research assistant retrieved data on demographics, anthropometrics [i.e. body mass index (BMI) at baseline and at the end of follow up], clinical features at diagnosis (e.g. stage of disease), administered therapy and treatment outcomes.

### Clinical Pathology Assays

Pre-treatment fasting glucose was measured on venous blood collected at the time of (histologically-confirmed) breast cancer diagnosis and previous to any form of cancer therapy. Blood samples were collected in standardized conditions claiming overnight fasting and time at blood drawing between 7 and 10 am.

Glucose concentrations were locally determined using a Cobas analyzer with Roche hexokinase reagent. Central laboratories at the Regina Elena National Cancer Institute (IRE) were certified to an international management systems standard called ISO 9001 (ISO 9001:2000/2008).

### Immunohistochemistry (IHC) and Silver in situ hybridization (SISH)

The immunohistochemical assessment of estrogen (ER) and progesterone (PgR) receptors as well as HER2, Ki-67 and p53 status was performed in formalin-fixed paraffin-embedded tissues using the monoclonal antibodies (MoAbs) 6F11, 1A6 (Menarini, Florence, Italy), the polyclonal antibody A0485 (PoAb, Dako, Milan, Italy), the MoAb MIB-1 (Dako) and the MoAb DO7 (Dako), respectively. Two micron-thick sections were stained with a streptavidin-enhanced immunoperoxidase technique (Supersensitive Multilink, Menarini) in an automated autostainer (Bond Max, Menarini) using a pH 6 citrate buffer antigen retrieval protocol for all the antibodies used throughout the study.

Assessment of the HER2 gene and Chromosome 17 status were performed by using a fully automated single color *in situ* hybridization assay based on a validated silver deposition technology (SISH, Inform HER2 DNA Probe; Inform Chr17 probe, Roche Diagnostics, Milan, Italy). The silver precipitation was visualized as a black dot in cell nuclei. The 100x oil immersion objective was used to score signals in all the neoplastic cells. SISH results were analyzed by using a light microscope (Nikon, Eclipse 55i) equipped with a software able to capture images (Eureka Interface System, Menarini, Firenze, Italy).

### Scoring criteria for IHC

ER, PgR, and p53 were considered positive when ≥10% of the neoplastic cells showed a distinct nuclear immunoreactivity whereas Ki67, based on the median value of our series, was regarded as high if ≥15% of the cell nuclei were immunostained. Bcl2 was recorded as positive when tumor cells exhibited a strong homogeneous cytoplasmic immunoreaction in more than 30% of neoplastic cells. HER2 IHC positivity was determined according to ASCO-CAP guidelines and revised according to the most recent update [36-37] [41-42] and was scored as follows: 0 (no staining or membrane faint incomplete staining in <10% tumor cells) and 1+ (faint incomplete membrane staining in <10% tumor cells) negative, 2+ (weak to moderate incomplete/complete staining in >10% tumor cells) unclearand 3+ (strong complete staining in >10% cells) positive. Tumors with a 2+ staining were defined negative or positive according to gene amplification. Evaluation of the IHC results was performed independently by two investigators blinded to all patient data.

### Scoring Criteria for SISH

According to the ASCO-CAP guidelines (36), tumors were defined as “non-amplified” (NA) by SISH when a HER2 gene copy number <6 was observed. Cases were defined as “amplified” (A) when SISH displayed a gene copy number >6. Polysomy 17 - intended as an increased CEP17 copy number (CEP17CN) – is considered to be present in BC when a mean number of ≥3 signals is shown.

### p53 Status: validation against DNA sequencing in a subset of 49 randomly selected breast cancer patients

Immunohistochemical p53 status was validated against gene sequencing in a randomly selected subset of 49 breast cancer patients from the 106 included in the present study. Patient selection was driven by a computer generated randomization list. The number of included patients resulting positive and negative at immunostaining were 30 and 19, respectively. The lab personnel involved in gene sequencing was blind to p53 status as assessed by immunohistochemistry.

### DNA extraction and Sequencing

DNA was extracted from 10 μ paraffin-embedded tumor tissue (PETT) sections after macrodissection using the DNA extraction kits QIAmp DNA kit (Qiagen-Explera, Jesi, Italy) according to the manufacturer's instructions. About 100-200 ng of genomic DNA was used in a polymerase chain reaction (PCR) to amplify the genomic region of TP53 harboring the higher mutational percentage, i.e., the hot spots 175, 245, 248, 249 and 273 codons within exons 5, 7 and 8. The following primers were used:

Forward-175: TCAACAAGATGTTTTGCCAACTGGCCAA;

Reverse-175: CATCGCTATCTGAGCAGCGCTCAT, For-248: GTTGGCTCTGACTGTACCACCATCCA

Rev-248: CTGGAGTCTTCCAGTGTGATGATGGT; For-273: TGGTAATCTACTGGGACGGA

Rev-273: CTCGCTTAGTGCTCCCTGGG

Sequence analysis was performed by Primm srl (MI). Collected data were evaluated throughout the the CHROMAS Analysis Software.

### Statistical Analyses

We examined distributions and computed descriptive statistics for all the variables of interest. We described the study participants' features and reported them through categories of pre-treatment fasting glucose and by p53 status. We used means and standard deviations for continuous data as well as frequencies and percentage values for categorical data. Existing differences between mean values were evaluated using the T-Student. We used the Pearson's Chi-squared test of independence (2-tailed) to assess the relationships between categorical variables.

We performed survival analyses using the Kaplan-Meier product-limit method and applied the Log-rank test to compare the survival curves by categories of pre-treatment fasting glycaemia. In the overall study population, time to disease progression was calculated as the interval between the date at first administration and (the date at) disease progression, last follow-up or death, whichever came first. Stratification by stage at diagnosis allowed for analyses separately including data from the neoadjuvant and adjuvant (early) and metastatic (advanced) setting, with disease free survival and progression free survival being the outcomes of interest, respectively. Survival curves were stratified by p53 staining pattern (p53 negative vs. positive) in the overall study population and subgroups defined by disease setting (early vs. advanced). The Cox proportional hazard model was used to further test the predictive role of fasting glucose on patient important outcomes in multivariate analyses. Age and BMI at cancer diagnosis were included as covariates. Based on recent evidence supporting the interaction between p53 status and BMI in cancer, an interaction term was added to the Cox model [26, 38] [43].

We considered p values less than 0.05 statistically significant. All statistical analyses were performed with the SPSS statistical software version 21 (SPSS inc., Chicago IL, USA).
